# The Risk of Acute Kidney Injury and Its Impact on 30-Day and Long-Term Mortality after Transcatheter Aortic Valve Implantation

**DOI:** 10.1155/2012/483748

**Published:** 2012-12-26

**Authors:** Katrin Gebauer, Gerhard-Paul Diller, Gerrit Kaleschke, Gregor Kerckhoff, Nasser Malyar, Matthias Meyborg, Holger Reinecke, Helmut Baumgartner

**Affiliations:** ^1^Division of Angiology, Department of Cardiovascular Medicine, University of Muenster, 48149 Muenster, Germany; ^2^Division of Adult Congenital and Valvular Heart Disease, Department of Cardiovascular Medicine, University of Muenster, 48149 Muenster, Germany

## Abstract

*Background*. Transcatheter aortic valve implantation (TAVI) is widely used in high risk patients (pts) with aortic stenosis. Underlying chronic kidney disease implicates a high risk of postprocedural acute kidney injury (AKI). We analyzed its occurrence, impact on hospital stay, and mortality. *Methods*. 150 consecutive pts underwent TAVI in our institution (mean age 81 ± 7 years; logistic EuroSCORE 24 ± 15%). AKI definition was a creatinine rise of 26.5 *μ*mol/L or more within 48 hours postprocedural. Ten patients on chronic hemodialysis were excluded. *Results*. AKI occurred in 28 pts (20%). Baseline creatinine was higher in AKI pts (126.4 ± 59.2 *μ*mol/L versus 108.7 ± 45.1 *μ*mol/L, *P* = 0.09). Contrast media use was distributed evenly. Both, 30-day mortality (29% versus 7%, *P* < 0.0001) and long-term mortality (43% versus 18%, *P* < 0.0001) were higher; hospital stay was longer in AKI pts (20 ± 12 versus 15 ± 10 days, *P* = 0.03). Predicted renal failure calculated STS Score was similar (8.0 ± 5.0% [AKI] versus 7.1 ± 4.0% [non-AKI], *P* = 0.32) and estimated lower renal failure rates than observed. *Conclusion*. AKI remains a frequent complication with increased mortality in TAVI pts. Careful identification of risk factors and development of more suitable risk scores are essential.

## 1. Introduction

Calcific aortic valve stenosis has become the most common acquired valve disorder with the highest prevalence in the 8th or 9th decade of life [1]. Comorbidities such as diabetes mellitus, stroke, coronary heart disease, peripheral artery disease, pulmonary disease, and renal impairment appear to markedly increase the risk of conventional valve replacement in the elderly and may limit the benefit of surgery in these patients [2]. Nevertheless, the outcome of untreated aortic stenosis is dismal once congestive heart failure, angina pectoris, or syncope occur. Therefore alternative, less invasive treatment options are needed. Since the first-in-man transcatheter aortic valve implantation (TAVI) by Cribier et al. in 2002 [3], more than 50000 procedures were performed worldwide and this intervention has become an accepted—assumingly less invasive—treatment alternative for high risk surgical patients. However, due the comorbidities in these patients, even TAVI is associated with a number of complications that may lead to impaired outcome. Their closer evaluation and the definition of risk factors as well as measures to reduce their occurrence are essential and require further research.

Acute kidney injury (AKI) is a well-known complication of angiography with the use of iodinated contrast media that accounts for a significantly prolonged hospital stay and worse in-hospital outcome [4]. Furthermore AKI has been shown to be an independent predictor of mortality [5–8]. The most important risk factor for AKI in patients undergoing standard heart catheterization is preexisting chronic kidney disease [9, 10]. Other risk factors include volume depletion, hemodynamic instability, and the use of nephrotoxic drugs.

TAVI requires the administration of contrast media and preexisting kidney disease is frequent in the currently treated patient population. However, the incidence of AKI, predictors of this complication, and its impact on outcome in patients undergoing TAVI have so far been poorly defined. 

Therefore, we sought to assess the incidence of AKI, search for its predictors, and analyze its impact on 30-day as well as midterm outcome in a sizeable group of consecutive patients undergoing TAVI. 

## 2. Materials and Methods

A total of 150 consecutive patients with symptomatic aortic stenosis who underwent TAVI in our institution because they were either not suitable for conventional surgical valve replacement or were considered at high operative risk by a multidisciplinary team including cardiologists and cardiac surgeons were included in this study. Ten out of 150 patients had been enrolled in a chronic dialysis program and were therefore excluded from analysis concerning AKI. Before intervention, all patients received left and right heart catheterization. According to the institution policy, written informed consent was obtained for treatment from each patient. Blood samples for hematology and serum chemistry were drawn one day prior to intervention and daily up to 72 hours after treatment. Patients with a previously impaired kidney function (estimated glomerular filtration rate, eGFR <60 mL/min/1,73 m²) received an intravenous prehydration protocol consisting of saline 0.9% with 1200 mg of n-acetyl-cysteine both 12 hours before and after the procedure. 

The vascular access site was evaluated by color-coded Doppler sonography and CT-angiography. Valve replacement was performed under general anaesthesia, except in one case which was done under local anaesthesia. All procedures were performed in the catheter laboratory using fluoroscopic guidance and nonionic isoosmolar contrast media iopromide (ultravist 370 (TM), Schering AG, Berlin, Germany) and transoesophageal echocardiography. The patients received either a 23 or 26 mm Edwards-Sapien valve prothesis. Patients not suitable for transfemoral TAVI because of poor vascular access underwent transapical TAVI.

### 2.1. Determination of Renal Function

Renal function at baseline and after 48 hours was determined from serum creatinine determined by the method of Jaffé. Since creatinine is known to be an insufficient marker of renal function but estimated glomerular filtration rate (eGFR) is considered most suitable we used the modification of diet in renal disease (MDRD) formula for calculation [11]:

(1) eGFR  (mL/min⁡/1,73 m2) =186×(creatinine,mg/dL)−1,154×(age,years)−0,203  (×  0,742  in  women).

### 2.2. Definition of Acute Kidney Injury

Acute Kidney Injury is divided into three stages by the Acute Kidney Injury Network: stage 1 is defined as a rise in serum creatinine ≥26.5 *μ*mol/L compared to baseline values or an increase in serum creatinine of more than or equal to 50% or a reduction in urine output as documented oliguria of less than 0.5 mL/kg per hour [12]. In this study AKI was defined as a 26.5 *μ*mol/L rise in serum creatinine 48 hours after procedure compared to baseline data drawn 24 hours before intervention. 

### 2.3. Prediction of Renal Failure and Mortality

Logistic EUROScore was calculated by the web-based system (http://www.euroscore.org/) [13] in advance and results taken for clinical decision making. STS Score was evaluated retrospectively for further analysis including the predicted risk of renal failure (http://209.220.160.181/STSWebRiskCalc261/de.aspx). Renal failure by STS Score is defined as an increase of serum creatinine >176.8 *μ*mol/L, a 50% or greater increase in serum creatinine over baseline preoperative value, or new requirement for dialysis.

### 2.4. Statistics

Differences in basic clinical characteristics between groups were tested by chi-square test for categorical and the ANOVA *F*-test for continuous variables. The *P* values for all of these tests are shown in the tables. Uni- and multivariate predictors of AKI were assessed by logistic regression analysis and odds-ratios (OR) are reported.

Univariate predictors of mortality during followup were analyzed by Cox regression and calculation of hazard rate ratios (HR) with 95% confidence intervals (95% CI). Multivariate analysis of mortality was performed by Cox regression analyses with potential covariates (adjusted HR). As covariates for adjustment, those parameters were chosen which were found to have a *P* value lower than 0.05 in univariate analyses of death. 

For all tests, *P*  values <0.05 were considered as significant. All statistical analyses were performed with PASW 18.0.0 for Windows.

## 3. Results

### 3.1. Baseline Data and Periprocedural Characteristics with Respect to Different Access Mode

The mean age in the patient group was 81 ± 7 years. In total, 96 patients received valve replacement via transfemoral (TF) and 54 patients via transapical (TA) approach. 91 (61%) of the patients were female. Patients with transapical approach were more frequently male and had significantly more underlying comorbidities such as hypertension, PAD, CHD, previous CABG, previous stroke, and impaired kidney function based on baseline serum creatinine measurement (see Table 1).

Ten patients (7%) had already been enrolled in a chronic dialysis program before intervention (5% of TF versus and 9% of TA patients, *P* = 0.006) and were therefore excluded from analysis concerning AKI.

The average amount of contrast media used in all patients was 147 ± 58 mL. Patients with transfemoral approach received with 160 ± 57 mL of contrast media a significantly greater amount than patients with transapical access who received 125 ± 53 mL (*P* < 0.0001). 

### 3.2. Acute Kidney Injury after Diagnostic Coronary Angiography

The rate of AKI after pre-TAVI diagnostic right and left heart catheterization in our patient population was 9.2% (*n* = 13). Only 4 patients (2.8%) developed AKI after both diagnostic coronary angiography and TAVI procedure (median 15 days between diagnostic catheterization and valve procedure). 

### 3.3. Acute Kidney Injury after TAVI

After exclusion of the ten patients who had already been enrolled in a chronic dialysis program before TAVI, 140 patients were left for the analysis concerning the occurrence of acute kidney injury. 

The number of patients developing acute kidney injury was 28 (20%).

Need for transient dialysis occurred in six out of these 28 pts. with AKI (21%). Two patients without AKI needed short-term dialysis (one patient was hemofiltrated due to low cardiac output and consecutive renal impairment, another patient acquired septic shock with renal failure). 

There was no significant difference regarding weight, height, baseline creatinine, and hemoglobin values in pts. who developed AKI after intervention compared to those who did not (see Table 2). Patients with AKI were significantly younger (79 ± 9 yrs versus 82 ± 6 yrs, *P* = 0.008) and had more frequently comorbidities such as hypertension and previous CABG whereas differences in peripheral arterial disease, cerebrovascular disease, CHD, and hypercholesterolemia did not reach statistical significance. 

Although baseline creatinine was slightly higher in the AKI group (126.4 ± 59.2 *μ*mol/L versus 108.7 ± 45.1 *μ*mol/L, resp.) this difference did not also reach statistical significance which could have been due to the sample size.

The amount of contrast media used during the procedure was also very similar between groups (147 ± 71 mL versus 148 ± 56 mL, *P* = 0.93). 

### 3.4. Impact of AKI on Hospital Stay and Survival

Mean hospital stay was significantly longer in pts. with AKI than in those without AKI (20 ± 12 days versus 15 ± 10 days, *P* = 0.03). 

Both, 30-day-mortality (29% versus 7%, *P* < 0.0001) and cumulative mortality after a median followup of 309 days were significantly higher in AKI patients (43% versus 18%, *P* < 0.0001).

**Figure 1:**
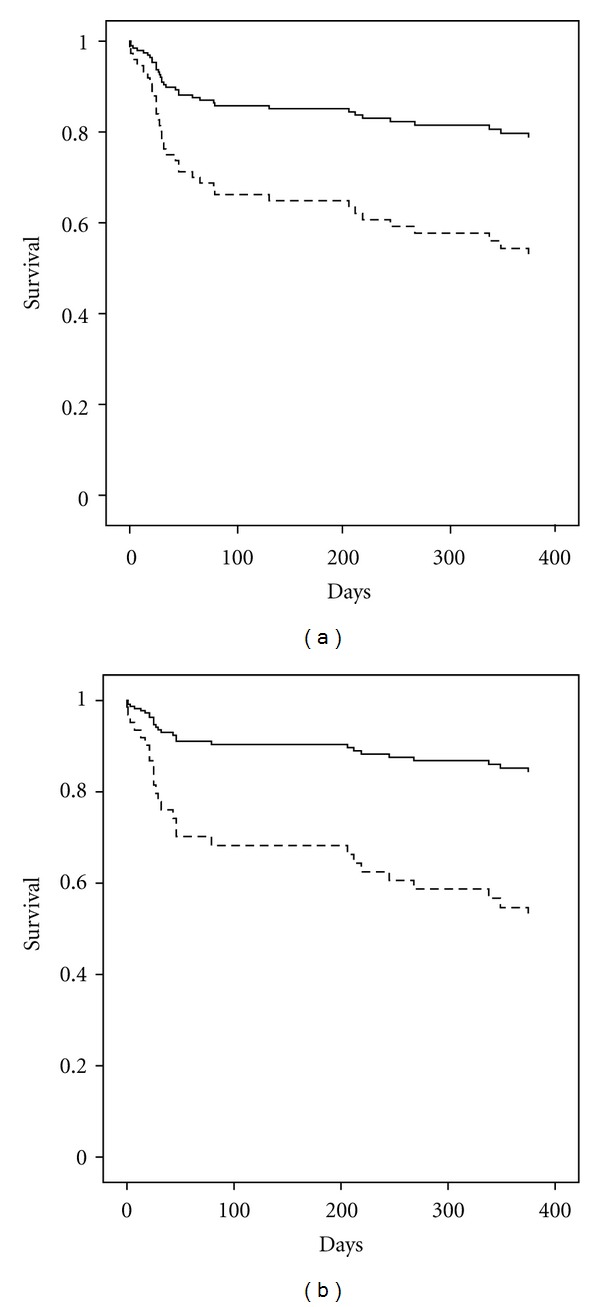
Cumulative survival in pts. with (dotted lines) and without AKI (solid lines). (a) Crude cumulative survival (days) (HRR 2.7, CI 1.34–5.41, *P* < 0.01). (b) Adjusted cumulative survival (days) (HRR 3.8, CI 1.37–10.37, *P* = 0.01); adjusted for age, diabetes, PAD, hypertension, previous myocardial infarction and CABG, left ventricular function, amount of contrast dye, baseline creatinine, and hemoglobin.

Crude and adjusted cumulative survival are shown in Figure 1. AKI was associated with significantly worse survival (HRR 2.7, CI 1.34–5.41, *P* = 0.006, Figure 1(a)). Mortality in AKI pts. was even higher (HRR 3.8, CI 1.37–10.37, *P* = 0.01, Figure 1(b)) after adjusting for risk factors (age, diabetes, PAD, hypertension, previous myocardial infarction and CABG, left ventricular dysfunction, amount of contrast dye, baseline creatinine, and hemoglobin).

### 3.5. Univariate and Multivariate Predictors of AKI

Predictors of AKI occurrence in univariate and multivariate regression analysis are shown in Tables 3 and 4. Of all included variables (age, diabetes, hypertension, PAD, previous CABG, myocardial infarction, left ventricular function, baseline creatinine, and hemoglobin and amount of contrast dye) only age was found to be significantly associated with AKI in univariate analysis and was detected as an independent predictor of AKI in multivariate analysis (OR 0.93, CI 0.87–0.99, *P* = 0.03). Including vascular access site in the model, transapical approach was also a significant predictor of AKI (OR 1.8, CI 1.85–18.4, *P* = 0.003).

### 3.6. EuroSCORE and STS Score

**Figure 2:**
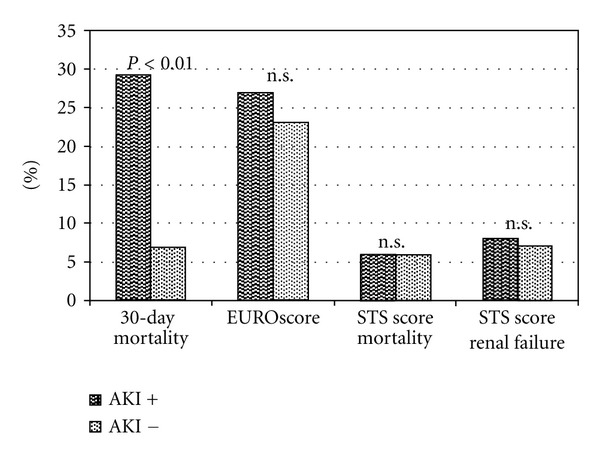
Morbidity and mortality with respect to access site and development of acute kidney injury (AKI): observed 30-day mortality and predicted renal failure and mortality by STS Score for pts. with and without AKI; AKI = acute kidney injury.

Neither EuroSCORE (27 ± 19% versus 23 ± 13, *P* = 0.18) nor STS Score (6.0 ± 3.5% versus 6.0 ± 3.4%, *P* = 0.97) predicted the marked difference in mortality rates between AKI and non-AKI pts (Figure 2).

When applying the STS Score renal failure definition to our patient population, 19 pts. (13.6%) developed renal failure. However, the predicted rate of renal failure by STS Score was only 7.3% (*P* = 0.023) for all pts. and therefore almost half of the observed rate. The predicted risk for renal failure based on the STS Score did not significantly differ between the TA and TF treatment groups (6.8 ± 3.5% versus 8.3 ± 5.1%, *P* = 0.054). The observed rate of AKI was however significantly higher in the TA group (31% versus 11%, *P* = 0.001) and exceeded thereby markedly the predicted rate of renal failure 1.6-fold in the TF and 3.7-fold in the TA pts. The amount of contrast dye in TA pts. was significantly less than in the TF pts. (125 mL versus 160 mL, *P* < 0.0001).

## 4. Discussion

Acute kidney injury after the use of iodinated contrast media in angiography is known to account for a number of adverse effects such as prolonged hospital stay [4] and to be an independent risk factor of mortality [7–10]. Patients undergoing TAVI procedures currently belong to an elderly population with numerous comorbidities. Several investigators have shown that AKI is a relatively frequent complication after TAVI and that it is associated with an increased mortality [14–17]. However it remains unclear which of the underlying comorbidities contribute most to the adverse outcome following AKI. Moreover, reliable predictors of AKI in this patient population still need to be defined. In particular, the value of risk scores developed for patients who undergo open heart surgery remains so far unknown. 

 In the present study, AKI occurred in 20% of the patients after TAVI with 4% requiring dialysis. This is within the range reported by other investigators. Nuis et al. [17] found very similar rates with 19% for AKI and 2% for temporary dialysis. While Bagur et al. [14] reported a lower rate of AKI (11%) and need of dialysis after TAVI (1.4%), Aregger et al. [18] and Kong et al. [19] found markedly higher rates of 28% and 7.4%, and 28.8% and 6%, respectively. One explanation for this wide variation of AKI rates could be that the average amount of contrast media used in these studies differed markedly, too. While it was 148 mL in the present study, Bagur et al. [14] with the lowest rate of AKI reported <100 mL. In contrast, Aregger et al. [18] with the higher rate of AKI used 242 mL on average. The amount of contrast media used during angiography is indeed considered one major risk factor for the development of AKI. Nevertheless, in the present study no significant difference in contrast media use could be found between AKI und non-AKI patients. The observation that AKI was not related to the amount of contrast used has also been reported by other groups [15, 16] suggesting that other factors may be more important for the development of renal impairment in the population currently undergoing TAVI. 

Prehydration in addition to intravenous n-acetyl-cysteine application prior to contrast media exposure is a well-known measure to reduce AKI rates in patients with renal impairment [8]. In our study patients with an eGFR <60 mL/min/1,73 m^2^ were treated with 1000 mL of saline 0.9% and 1200 mg of n-acetyl-cysteine which may have prevented more patients from experiencing AKI than without these protective measures. Nevertheless, this prevention strategy in general and even more in this specific patient population is not effective enough to thoroughly avoid occurrence of AKI. It can however not be excluded that this therapy had impact on our results. Therefore hydration therapy may have contributed to the nonsignificant association between baseline creatinine and acute kidney injury risk.

Comparing AKI rates and patients after diagnostic and TAVI catheterization no correlation between AKI after diagnostic and valve procedure could be seen implying the lack of a patient-inherent predisposition for AKI occurrence after exposure to contrast media. 

This finding complies with the results of Van Linden et al. [20] who stated that early contrast media exposure (1–7 days) by cardiac catheter or CT-Scan did not increase the risk of AKI or RRT.

In this context it should be kept in mind that after cardiac surgery without any use of contrast media, the rate of AKI can also reach up to 30% with 1% requiring dialysis treatment [21, 22]. Bagur et al. [14] reported a 25% incidence of AKI after surgical aortic valve replacement in patients with preprocedural chronic kidney disease compared to 12% in patients undergoing TAVI.

 The markedly adverse effect of the occurrence of AKI on the outcome of TAVI underlines the importance of identifying predictors of this complication as well as appropriate measures for its prevention. In the present study, 30-day mortality and midterm mortality were as high as 29% and 43% in patients with AKI compared to only 7% and 18% in those who did not develop this complication. The difference in survival was even more pronounced when adjusting for differences in baseline characteristics. Similar findings have been reported by other investigators [14–17, 23]. Despite these rather consistent findings with regard to incidence of AKI after TAVI and its adverse impact on outcome, the data with regard to risk prediction and options for prevention of AKI remain controversial. 

It appears obvious that preprocedural chronic kidney disease should be a major risk factor for the development of postprocedural AKI. Sinning et al. [16] indeed showed impaired renal function with moderately elevated serum creatinine values before intervention and AKI occurrence unrelated to the amount of contrast media to be the strongest predictors of 1-year mortality among TAVI patients. Although baseline creatinine in the present study was slightly higher in the AKI group (126.4 ± 59.2 *μ*mol/L versus 108.7 ± 45.1 *μ*mol/L, resp.) this difference did not reach statistical significance. This may be due to small sample size and thus lack of statistical power. As a matter of fact, preinterventional serum creatinine was found to be a significant predictor of AKI only in the study by Elhmidi et al. [15] whereas several other studies could not confirm this observation [14, 15, 17, 19]. Paradoxically, younger age turned out to be the only independent preprocedural risk factor for the development of AKI in the present study. This observation must be seen with caution. To qualify for TAVI instead of conventional surgery, younger patients must assumingly have been markedly sicker than older patients. It is therefore likely that this observation is due to confounding factors. Patients with AKI more frequently had hypertension and previous bypass surgery. Hypertension was also found to be a predictor of AKI in other studies [14, 19]. Without consistency, peripheral artery disease [15, 16], previous myocardial infarction [17], chronic obstructive pulmonary disease [14], systemic inflammatory response [16, 17], residual aortic regurgitation [16], and periprocedural red blood cell transfusion [14, 15, 19] have been reported to predict AKI after TAVI. In accordance with Kong et al. [19], transapical TAVI was found to be associated with a higher risk of AKI in the present study. Although this could be partially due to the worse baseline characteristics of these patients, transapical access remained a significant predictor after consideration of such differences. Whether the more invasive nature of this approach, higher bleeding rates and requirement for blood cell transfusion account for this difference remains to be shown. Nuis et al. [17] found the logistic EuroSCORE to predict AKI. This could not be confirmed in the present study. In addition, similar to previous reports, the observed 30-day mortality was markedly lower than predicted by the logistic EuroSCORE (11% versus 24%) whereas the STS score was indeed lower (6%). This is in agreement with the observation of Piazza et al. [24] who found lower estimates of operative mortality by the STS Score stating that this scoring system has suboptimal discriminatory power and calibration for TAVI patients. STS score as a surgical risk algorithm obviously omits several risk factors in the TAVI population leading to different patient selection and thus mortality rates. The present study also demonstrates that STS score for prediction of renal failure has little value for prediction of renal failure after TAVI. This underlines once more the importance of developing appropriate scores for the risk of death as well as of the risk of renal failure and other complications in patient populations currently treated with TAVI. 

 In addition to preexisting factors, hemodynamic instability with consecutive extreme hypotension caused by rapid pacing, balloon valvuloplasty, and prosthesis deployment during TAVI may account for a significantly higher risk of AKI in patients undergoing TAVI compared to simple angiography or PCI. Arteriosclerotic microembolism may also contribute to temporary deterioration of kidney function. This must be considered when developing measures to reduce the occurrence of AKI after TAVI.

 This study has several limitations. Although the data were collected prospectively in consecutive patients undergoing TAVI, the analysis with regard to incidence and predictors of AKI was performed retrospectively. Potentially relevant factors such as red blood cell transfusion, after procedure thrombocytopenia and hemoglobin drop, procedure time, hemodynamic complication or the use of angiotensin converting enzyme inhibitors, and/or angiotensin receptor blockers could not be evaluated. Although the study comprised a sizeable number of TAVI patients, it reflects a single-center experience only and a much larger population is required to perform extensive multivariate analyses in order to better identify risk factors for the development of AKI with relevant impact on the decision making in clinical practice.

## 5. Conclusion

Development of AKI is frequent in patients undergoing TAVI. Its occurrence does not appear to be primarily related to the amount of contrast dye used. Furthermore, STS Score does not reliably predict renal failure in this patient population. The occurrence of AKI markedly increases hospital stay as well as 30-day and midterm mortality even after consideration of the baseline risk profile. Thus, improvements in predicting the risk of AKI after TAVI as well as effective measures to reduce the rate of this complication would be essential.

## Figures and Tables

**Table 1:** Baseline data and periprocedural characteristics with respect to different access modes for valve replacement.

	Total	Transfemoral	Transapical	*P *
	*n* = 150	*n* = 96	*n* = 54	
Women, *n* (%)	91 (61)	66 (69)	25 (46)	**0.006**
Age, mean ± s.d., years	81 ± 7	82 ± 6	80 ± 7	0.2
Hypertension, *n* (%)	115 (77)	69 (72)	46 (85)	**<0.0001**
Endstage renal failure, *n* (%)	10 (7)	5 (5)	5 (9)	0.06
Hypercholesterolemia, *n* (%)	51 (34)	31 (32)	20 (37)	0.27
Diabetes, *n* (%)	42 (28)	25 (26)	17 (31)	0.56
Peripheral occlusive disease, *n* (%)	37 (25)	10 (10)	27 (50)	**<0.0001**
Coronary heart disease, total, *n* (%)	79 (53)	41 (43)	34 (63)	**<0.05**
(1) vessel disease	25 (17)	13 (14)	12 (22)	
(2) vessel disease	20 (13)	14 (15)	6 (11)	
(3) vessel disease	34 (23)	14 (15)	20 (37)	
Previous myocardial infarction, *n* (%)	36 (24)	22 (23)	14 (26)	0.42
Previous CABG, *n* (%)	34 (23)	15 (16)	19 (35)	**<0.0001**
Previous stroke, *n* (%)	14 (9)	7 (7)	7 (13)	**0.024**
Baseline creatinine, mean ± s.d., *μ*mol/L	133.5 ± 109.6	116.7 ± 73.4	162.7 ± 150.3	**0.013**
GFR, mean ± s.d., mL/min/1,73 m^2^	53 ± 22	55 ± 21	49 ± 22	0.11
Baseline hemoglobin, mean ± s.d., mmol/L	7.39 ± 1.06	7.39 ± 1.06	7.39 ± 0.99	0.8
Hemoglobin 24 hours after intervention, mean ± s.d., mmol/L	6.27 ± 0.81	6.27 ± 0.74	6.21 ± 0.93	0.53
Amount contrast dye, mean ± s.d., mL	147 ± 58	160 ± 57	125 ± 53	**<0.0001**
STS Score renal failure, % ±s.d.	7.3 ± 4.2	6.8 ± 3.5	8.3 ± 5.1	0.054
STS Score mortality, % ±s.d.	6.3 ± 3.5	5.7 ± 2.7	7.1 ± 4.5	**0.024**
EUROScore, % ±s.d.	24 ± 14	22 ± 13	28 ± 16	**0.02**

All values are expressed as mean ± s.d. or *n* (%).

**Table 2:** Parameters associated with acute kidney injury (AKI).

	All (*n* = 140*)	With AKI (*n* = 28)	Without AKI (*n* = 112)	*P* value
Age (years) ± s.d.	82 ± 7	79 ± 9	82 ± 6	**0.008**
Weight (kg) ± s.d.	74 ± 16	76 ±17	74 ± 16	0.535
Height (cm) ± s.d.	167 ± 9	169 ± 9	167 ± 8	0.136
Baseline creatinine (*μ*mol/L) ± s.d.	114.9 ± 53	126.4 ± 59.2	108.7 ± 45.1	0.093
GFR (mL/min/1,73 m^2^) ± s.d.	55 ± 20	51 ± 17	57 ± 20	0.16
Baseline hemoglobin (mmol/L) ± s.d.	7.45 ± 1.06	7.26 ± 1.24	7.45 ± 0.99	0.35
Hemoglobin 24 hours after intervention, mean ± s.d., mmol/L	6.27 ± 0.81	6.21 ± 1.06	6.33 ± 0.74	0.56
Amount of contrast media (mL) ± s.d.	148 ± 59	147 ± 71	148 ± 56	0.93
Diabetes, *n* (%)	38 (27)	10 (36)	28 (25)	0.19
Hypertension, *n* (%)	108 (77)	24 (86)	84 (75)	**0.007**
PAD, *n* (%)	34 (24)	7 (25)	27 (24)	0.45
Previous stroke, *n* (%)	11 (8)	2 (7)	9 (8)	0.75
Previous CABG, *n* (%)	32 (23)	9 (32)	23 (21)	**0.02**
Previous MI, *n* (%)	34 (24)	9 (32)	25 (22)	0.06
CHD, *n* (%)	74 (53)	20 (71)	54 (48)	0.6
Hypercholesterolemia, *n* (%)	48 (34)	10 (36)	38 (34)	0.73
STS Score renal failure, % ±s.d.	7.3 ± 4.2	8.0 ± 5.0	7.1 ± 4.0	0.32
STS Score mortality, % ±s.d.	6.0 ± 3.4	6.0 ± 3.5	6.0 ± 3.4	0.97
EUROScore, % ±s.d.	24 ± 15	27 ± 19	23 ± 13	0.18

All values expressed as mean ± s.d. or *n* (%); *10 pts. on chronic dialysis excluded.

**Table 3:** Univariate predictors of acute kidney injury.

	OR	CI 95%	*P *
Age	0.92	0.87–0.98	**0.012**
Diabetes	1.6	0.66–3.83	0.3
Hypertension	2.0	0.64–6.26	0.23
PAD	1.05	0.4–2.74	0.92
CABG	1.8	0.73–4.58	0.2
MI	1.65	0.66–4.09	0.28
Moderately reduced LV function*	1.63	0.56–4.7	0.37
Severely reduced LV function^#^	1.54	0.44–5.32	0.5
Baseline creatinine	1.92	0.96–3.83	0.06
Baseline hemoglobin	0.89	0.69–1.14	0.35
Amount contrast dye	1.0	0.99–1.01	0.93

*Moderately impaired left ventricular function (EF = 49–35%), ^#^severely impaired left ventricular function (EF < 35%); CI: Confidence interval for Exp (B) 95%.

**Table 4:** Multivariate predictors of acute kidney injury.

	OR	CI 95%	*P *
Age	0.93	0.87–0.99	**0.05**
Diabetes	1.3	0.48–3.6	0.6
Hypertension	2.0	0.54–7.41	0.3
PAD	0.76	0.25–2.3	0.62
CABG	1.49	0.5–4.4	0.48
MI	1.08	0.34–3.42	0.89
Moderately reduced LV function*	1.85	0.58–5.92	0.3
Severely reduced LV function^#^	1.0	0.23–4.3	0.9
Baseline creatinine	2.0	0.89–4.54	0.1
Baseline hemoglobin	0.86	0.66–1.14	0.3
Amount contrast dye	1.0	0.99–1.01	0.6
Constant	44.38		0.3

*Moderately impaired left ventricular function (EF = 49–35%), ^#^severely impaired left ventricular function (EF < 35%); CI: Confidence interval for Exp (B) 95%.

## Abbrevations

AKI: Acute kidney injury
AKIN: Acute kidney injury network
CABG: Coronary artery bypass graft
CHD: Coronary heart disease
EUROScore: European System for cardiac operative risk evaluation
GFR: Glomerular filtration rate
MI: Myocardial infarction
PAD: Peripheral arterial disease
TA: Transapical
TAVI: Transcatheter aortic valve implantation
TF: Transfemoral
STS Score: Society of Thoracic Surgeons Score.
